# CT Features of Sickle Cell Disease-Associated Pulmonary Hypertension

**DOI:** 10.3390/jcm15197611

**Published:** 2026-10-01

**Authors:** Nouran Waleed Molla, Sarah Sewaralthahab, Razan Abdulrahman Alotaibi, Abdulaziz Abdulrahman Alymni, Waad Mohammed Abu Nakha, Naif Adeeb Alquwayi, Khalid Abdulrahman Abdulaziz, Noof Abdulaziz Alsaigh

**Affiliations:** 1 Department of Radiology and Medical Imaging, College of Medicine, King Saud University, Riyadh 11451, Saudi Arabia; 2 Department of Medicine, College of Medicine, King Saud University, Riyadh 11451, Saudi Arabia; ssewar@ksu.edu.sa; 3 College of Medicine, King Saud University, Riyadh 11451, Saudi Arabia442100146@student.ksu.edu.sa (N.A.A.); 442200737@student.ksu.edu.sa (N.A.A.)

**Keywords:** sickle cell disease, pulmonary hypertension, computed tomography, pulmonary artery diameter, septal flattening, right ventricular hypertrophy, pleural effusion, pulmonary vascular remodeling

## Abstract

**Background/Objectives:** Sickle cell disease (SCD) frequently leads to pulmonary hypertension (PH), affecting 6–11% of patients and increasing mortality. Computed tomography (CT) can assess pulmonary vascular changes, but its role in SCD-associated PH remains unclear. This retrospective study aims to evaluate CT findings in adults with SCD-associated PH and their association with disease severity as assessed by Transthoracic Echocardiography (TTE) and right heart catheterization (RHC). **Methods:** We conducted a retrospective cohort study at a tertiary center in Riyadh, reviewing CT scans from 2015 to 2024. Adults with confirmed SCD were included if PH was suggested by echocardiography or confirmed by RHC. CT findings, including pulmonary artery diameter and parenchymal abnormalities, were analyzed alongside demographic and clinical data using Fisher’s exact tests with FDR correction for multiple comparisons. **Results:** Eighteen adults were included (mean age 35.9 ± 10.8 years; 50% male). Of the 18 patients, five (27.8%) had RHC-confirmed PH, whereas the remaining 13 (72.2%) were included based on TTE findings suggestive of PH without RHC confirmation. Echocardiographic probability data were available for 17 patients, among whom intermediate probability was the most frequent category (11/17, 64.7%). CT demonstrated PH signs in 55.6% of patients. Mean pulmonary artery diameter was 2.90 ± 0.60 cm, greatest in patients assessed by both TTE and RHC (3.61 ± 0.58 cm). The most frequent CT findings were cardiomegaly (44.4%) and pleural effusion (38.9%). Using Fisher’s exact test, no individual CT feature was significantly associated with PH severity after correction for multiple comparisons; the presence of any CT sign of PH showed a nominal association with severity (exact *p* = 0.042) that was no longer significant after correction for multiple comparisons (q = 0.33). CT classified more patients as mild or moderate, but fewer as severe than TTE; neither septal flattening nor RV hypertrophy was significantly associated with severe PH on exact testing (*p* = 0.10 and *p* = 0.20, respectively, on CT-based assessment), and these associations should be considered exploratory given the small sample size. **Conclusions:** CT identifies vascular, cardiac, and parenchymal abnormalities, but does not replace TTE and RHC in evaluating SCD-associated PH.

## 1. Introduction

Sickle cell disease (SCD) is an inherited blood disorder characterized by the production of irregular hemoglobin (hemoglobin S), leading to red blood cells assuming a crescent or sickle shape. This condition affects approximately 7.74 million people globally, with a notably higher prevalence among individuals of African, Mediterranean, Middle Eastern, and South Asian descent. The underlying cause of SCD is a mutation in the gene that produces hemoglobin, a protein essential for oxygen transport in red blood cells. This mutation results in the production of abnormal hemoglobin S, which causes red blood cells to become rigid and sticky [1,2,3,4]. Consequently, individuals with SCD experience various health challenges, including chronic pain, extreme fatigue, anemia, and increased susceptibility to infections [5]. The structural change in hemoglobin creates a scenario for the development of SCD-related complications like hemolytic anemia and increased thromboembolic risks, all of which contribute to the pathophysiology of pulmonary hypertension (PH) [6].

In Saudi Arabia, SCD represents a significant public health concern, with marked regional variation in prevalence. The highest rates have been reported in the Eastern Region, followed by the southern regions, particularly Asir and Jazan, whereas Hail has demonstrated the lowest rates of both SCD and sickle cell trait (SCT) [7]. These regional variations highlight the need for targeted prevention and screening strategies.

PH is a common complication in patients with SCD. Clinical symptoms of PH include shortness of breath, fatigue, chest pain, and swelling in the limbs. Physical examination may uncover signs of right ventricular dysfunction, such as a pronounced second heart sound, a fourth heart sound on the right side, right ventricular enlargement, and a right parasternal heave. These clinical findings provide crucial information on the structural and functional changes in the cardiovascular system, assisting in diagnosing PH and helping to shape appropriate treatment strategies [5].

The pathogenesis of PH in SCD is likely multifactorial [8]. A key contributor is chronic intravascular hemolysis, which releases cell-free hemoglobin into the plasma, leading to nitric oxide (NO) scavenging and endothelial dysfunction [9,10,11,12]. Additional mechanisms include pulmonary thromboembolism [13,14,15]. Together, these processes highlight the complex interplay of hemolytic, enzymatic, thrombotic, and vascular remodeling pathways in SCD-associated PH.

SCD also predisposes sickle cell patients to pulmonary arterial hypertension (PAH), which is a critical condition that can lead to extended morbidity and death. Its pathogenesis, clinical manifestations, and progression must be understood for better patient management [6]. The European Society of Cardiology (ESC) Guidelines define PAH as a mean pulmonary artery pressure greater than 20 mmHg (mPAP), which occurs in about 6–11% of the SCD population [16]. Even though mPAP values in this population are relatively low, the presence of sickle-related PH confers extremely high mortality, making the need for stratified management to control disease progression, poor health outcomes, and death necessary [16].

The reported prevalence of PH in patients with SCD varies considerably depending on the diagnostic modality and study population. Direct right heart catheterization (RHC) studies estimate a prevalence of approximately 6–11% [16,17]. In contrast, echocardiographic screening studies report higher rates ranging from 10% to 33% [6]. In the United States, the prevalence of SCD-associated PH has been reported at 32% [9], while a study from Eastern Saudi Arabia (Al-Ahsa region) reported a prevalence of 37.1% based on echocardiographic assessment [18]. Autopsy studies suggest an even greater burden, demonstrating PH-related pathological changes in up to 75% of affected patients [6,19].

These findings highlight the substantial burden of PH in patients with SCD and underscore the challenges in accurately determining its true prevalence, owing to variations in disease severity, study populations, and diagnostic approaches.

PH represents a major prognostic determinant in SCD, with even mild elevations in pulmonary artery pressure associated with increased morbidity and mortality [17]. Despite relatively modest increases in pulmonary artery pressure, PH contributes substantially to adverse outcomes in this population [6]. Chronic hemolysis plays a central role in PH pathogenesis, and disease-modifying therapies such as hydroxyurea and elevated fetal hemoglobin levels appear to confer protective effects [20]. Current recommendations discourage routine echocardiographic screening for PH in asymptomatic patients with SCD. Instead, further diagnostic evaluation should be guided by clinical signs, symptoms, or other findings that raise suspicion for PH. This highlights the importance of appropriate patient selection for further diagnostic evaluation [21].

Transthoracic Echocardiography (TTE) is widely used as a noninvasive screening tool for PH in patients with SCD; it allows assessment of cardiac structural changes and estimation of pulmonary artery systolic pressure through measurement of the tricuspid regurgitant jet velocity (TRV), but its diagnostic performance in this population remains limited. In a cohort with a PH prevalence of approximately 6%, a TRV threshold of ≥2.5 m/s demonstrated a low positive predictive value, with substantial variability in sensitivity and specificity across proposed cutoff values [22]. Although combining echocardiographic findings with biomarkers or functional measures may improve predictive performance, echocardiography alone remains insufficient for accurate risk stratification. There is no consensus regarding optimal screening strategies or selection of patients who would benefit from confirmatory right heart catheterization, the diagnostic gold standard [22]. Sensitivity and specificity also vary significantly, with TRV ≥ 2.5 m/s demonstrating high sensitivity but poor specificity, while higher thresholds improve specificity at the expense of sensitivity [23].

The role of computed tomography (CT) in the evaluation of PH associated with SCD remains incompletely defined, while high-resolution computed tomography (HRCT) has shown limited ability to detect PH-related abnormalities in some SCD cohorts, with minimal radiological differences between patients with and without PH [24].

PH is frequently diagnosed late due to nonspecific symptoms and overlap with other cardiopulmonary conditions [25]. Using established criteria, PH has been identified in 6.0–10.4% of adults with SCD, supporting the need for a detailed and structured diagnostic approach [17]. Imaging plays a central role in PH evaluation, including detection, etiological assessment, and disease monitoring. Imaging modalities used in PH assessment range from chest X-ray (CXR) as an initial investigation to computed tomography pulmonary angiography (CTPA) for detailed assessment of pulmonary vascular anatomy. Emerging techniques such as single-photon emission computed tomography (SPECT) and dual-energy CT (DECT) are under evaluation, though their definitive roles remain incompletely defined [26]. CT offers rapid acquisition, high spatial resolution, and detailed assessment of pulmonary vasculature, lung parenchyma, and cardiovascular remodeling. While CT does not replace invasive hemodynamic assessment, its strength lies in identifying underlying etiologies and structural changes associated with disease severity [26]. Advanced CT techniques further demonstrate potential as noninvasive surrogate markers of pulmonary hemodynamics and therapeutic response, supporting their emerging role in PH evaluation [27]. DECT has been associated with imaging patterns including ground-glass opacities, perfusion defects, infarct-related changes, and mosaic attenuation; however, only half of patients with abnormal DECT findings demonstrate corresponding evidence of PH on echocardiography or cardiopulmonary exercise testing. CT-based studies have also reported enlargement of the pulmonary trunk and arterial branches in patients with SCD compared with controls, with strong concordance between automated and manual measurements [28].

While progress has been made in understanding PH associated with SCD, major gaps remain. Most available studies are either cross-sectional or retrospective in nature, which uncouples treatment from outcome analysis [16,17]. Few studies have proposed targeted screening strategies or non-invasive diagnostic approaches [6,17,29]. SCD-associated PH warrants dedicated study for three reasons: its pathogenesis is mechanistically distinct from most other secondary PH etiologies, driven by chronic hemolysis, nitric oxide scavenging, endothelial dysfunction, and a chronic thrombotic tendency rather than the pressure- or volume-overload mechanisms that dominate other causes of secondary PH; SCD carries a substantial, regionally concentrated disease burden in Saudi Arabia; and while CT’s general role in PH evaluation is increasingly described, CT findings specific to SCD-associated PH remain comparatively sparse in the literature. This last point is the gap the present study addresses. This retrospective cohort study aims to characterize CT imaging features in adults with SCD and either echocardiographic suspicion of PH or hemodynamically confirmed PH and determine whether CT-derived measurements and findings are associated with disease severity as assessed by TTE and RHC.

## 2. Materials and Methods

This was a retrospective cohort study of adults with SCD who had either echocardiographic findings suggesting PH or hemodynamically confirmed PH by RHC at a tertiary center in Riyadh, Saudi Arabia. Medical records of patients who underwent chest CT imaging between 2015 and 2024 were retrieved from the hospital electronic system, Cerner PowerChart (v2018.01; Cerner Corporation, Kansas City, MO, USA).

CT scans were included based on the following criteria: age ≥ 18 years, a confirmed diagnosis of SCD, either PH suggested by TTE or PH confirmed by RHC, and cases with available CT imaging from 2015 to 2024. CT examinations were acquired using either Siemens CT systems (Siemens Healthineers, Forchheim, Germany) or GE CT systems (GE HealthCare, Waukesha, WI, USA) and reviewed using Centricity PACS (v7.0). CT scans were excluded if there was a history of primary pulmonary disease, primary vascular disease, congenital heart disease, autoimmune disease, or acute pulmonary embolism.

CT findings were initially extracted from the original clinical reports issued by the reporting radiologists during routine clinical care. All CT examinations were subsequently reviewed independently by a second radiologist with six years of experience, who was blinded to the original radiology reports and to the TTE and RHC findings. The second reader assessed all prespecified CT features, including mosaic attenuation, right-sided cardiac enlargement, interventricular septal flattening, and cardiomegaly. The second read was performed by a single radiologist, fully blinded to the original clinical reports and to the TTE and RHC findings. No formal interobserver agreement statistic was calculated. No disagreements were encountered between the second read and the original reports.

The retrieved CT scans underwent multiple filtering phases. First, duplicate scans were removed. For patients with multiple CT scans, only the most recent scan that was closest to the date of TTE and/or RHC, if available, was included in the analysis.

CT acquisition and image assessment: CT examinations were performed according to the institutional chest CT protocols applicable at the time of each examination, including CTPA, HRCT, and routine chest CT. A thin-section technique was used throughout, typically at 120 kV, with slice thickness and reconstruction parameters differing by clinical protocol. HRCT examinations used thin sections of approximately 0.625–0.65 mm acquired at inspiration, with both soft-tissue and lung reconstructions. For CTPA examinations, thin-section acquisition and reconstruction were used, with pulmonary arterial enhancement optimized using a timing-bolus or automated bolus-tracking technique. Routine contrast-enhanced chest CT examinations were performed with intravenous contrast administered at 3–4 mL/s and an acquisition delay of 40 to 70 s, with sagittal and coronal reformats. Non-contrast HRCT examinations were performed during inspiration without intravenous contrast.

For CT image assessment, predefined criteria were used to evaluate each imaging feature. Pulmonary artery diameter (PAD) was measured on axial CT images at the level of the main pulmonary artery bifurcation, with the main pulmonary trunk and ascending aorta visualized in the same imaging plane. The maximal diameter of the main pulmonary artery was measured from outer wall to outer wall, perpendicular to the long axis of the vessel, to minimize measurement error related to oblique orientation. Main pulmonary artery enlargement was defined as a main pulmonary artery diameter ≥ 30 mm on CT [30]. Cardiomegaly was defined as a cardiothoracic ratio > 0.50 on axial CT images, calculated as the maximal transverse cardiac diameter divided by the maximal transverse thoracic diameter [31]. Right atrial enlargement was defined as a transverse right atrial diameter ≥65 mm in women and ≥70 mm in men [32]. Right ventricular (RV) hypertrophy was defined as right ventricular outflow tract wall thickness ≥6 mm [30]. Interventricular septal flattening was assessed by abnormal septal configuration, with a septal angle ≥ 140° considered abnormal [30]. Mosaic attenuation was defined as a geographic patchwork of lung regions with differing attenuation on inspiratory CT [33]. Pulmonary edema was defined as CT evidence of interstitial and/or alveolar fluid accumulation, manifested by ground-glass opacities and interlobular septal thickening, with or without associated pleural effusion [34]. Pulmonary fibrosis was defined by architectural distortion and volume loss, with CT features including traction bronchiectasis or bronchiolectasis and/or honeycombing [33]. Pleural effusion was defined as an abnormal fluid-attenuation collection within the pleural space [33]. Pericardial effusion was identified as fluid within the pericardial space on CT, with CT allowing assessment of its location and size [35].

Because no validated criteria are currently available for classifying PH severity based on CT findings, we used a study-defined exploratory classification based on the number of CT features of vascular and cardiac remodeling: the presence of one feature was categorized as mild, two features as moderate, and three features as severe. This classification was developed for the purposes of the present analysis and was not intended to represent a validated CT severity grading system or to be equivalent to echocardiographic assessment of PH severity. Patients without any of the qualifying CT features were considered to have no CT signs suggestive of PH and were not assigned a CT-based severity category. Consequently, the exploratory CT severity analysis was restricted to the 10 patients who demonstrated at least one qualifying CT feature.

Sickle cell disease was then confirmed based on hemoglobin electrophoresis results, with hemoglobin S (HbS) levels > 50% considered diagnostic. For patients with confirmed SCD, TTE and RHC reports, when available, were reviewed to classify PH as echocardiographically suggested or hemodynamically confirmed.

For cohort ascertainment, echocardiographic suspicion of PH was operationally defined as a tricuspid regurgitation (TR) jet velocity ≥ 2.8 m/s and/or an estimated right ventricular systolic pressure (RVSP) ≥ 35 mmHg. These values represent the lower limits of abnormality in the American Society of Echocardiography guideline (ASE) reference table [36] and were used as screening thresholds for patient selection; they were not considered evidence of hemodynamically confirmed PH. Although reference [36] was published in 2025, the TR-jet and RVSP thresholds it specifies have not changed materially across recent iterations of the ASE guideline; we therefore applied these criteria retrospectively to echocardiography reports spanning the full 2015–2024 study period and cite the 2025 publication as the current definitive reference for these definitions.

Assessment of PH probability based on TTE was performed according to the TR jet velocity, with low probability defined as <2.8 m/s, intermediate probability as 2.8–3.4 m/s, and high probability as >3.4 m/s. PH severity based on RVSP on TTE was assessed according to criteria derived from the ASE, with mild severity defined as 35–49 mmHg, moderate as 50–69 mmHg, and Severe as ≥70 mmHg. For patients who underwent RHC, catheterization reports were reviewed, and only patients with confirmed PH were included in the final study cohort.

### Statistical Analysis

Continuous variables are presented as mean ± standard deviation and categorical variables as frequency (%). Associations between categorical CT features and PH severity category, and between individual CT features and severe PH, were assessed using Fisher’s exact test; for the three-category (mild/moderate/severe) comparisons, the Freeman–Halton extension of Fisher’s exact test was used, since several contingency tables had expected cell counts below 5, for which the chi-square approximation is unreliable. Exact odds ratios and 95% confidence intervals are reported for the 2 × 2 severe-versus-non-severe comparisons. Given the number of comparisons performed relative to the cohort size, a Benjamini–Hochberg false discovery rate (FDR) correction was applied within each family of comparisons, and FDR-adjusted q-values are reported alongside unadjusted *p*-values. Correlations between ordinal and continuous measures (e.g., pulmonary artery diameter, CT signs of PH, and PH severity) were assessed using Kendall’s tau-b. All tests were two-sided, and all associations—including those with *p* < 0.05—were interpreted as exploratory given the small sample size. Analyses were performed using IBM SPSS Statistics (version 26).

## 3. Results

**Figure 1:**
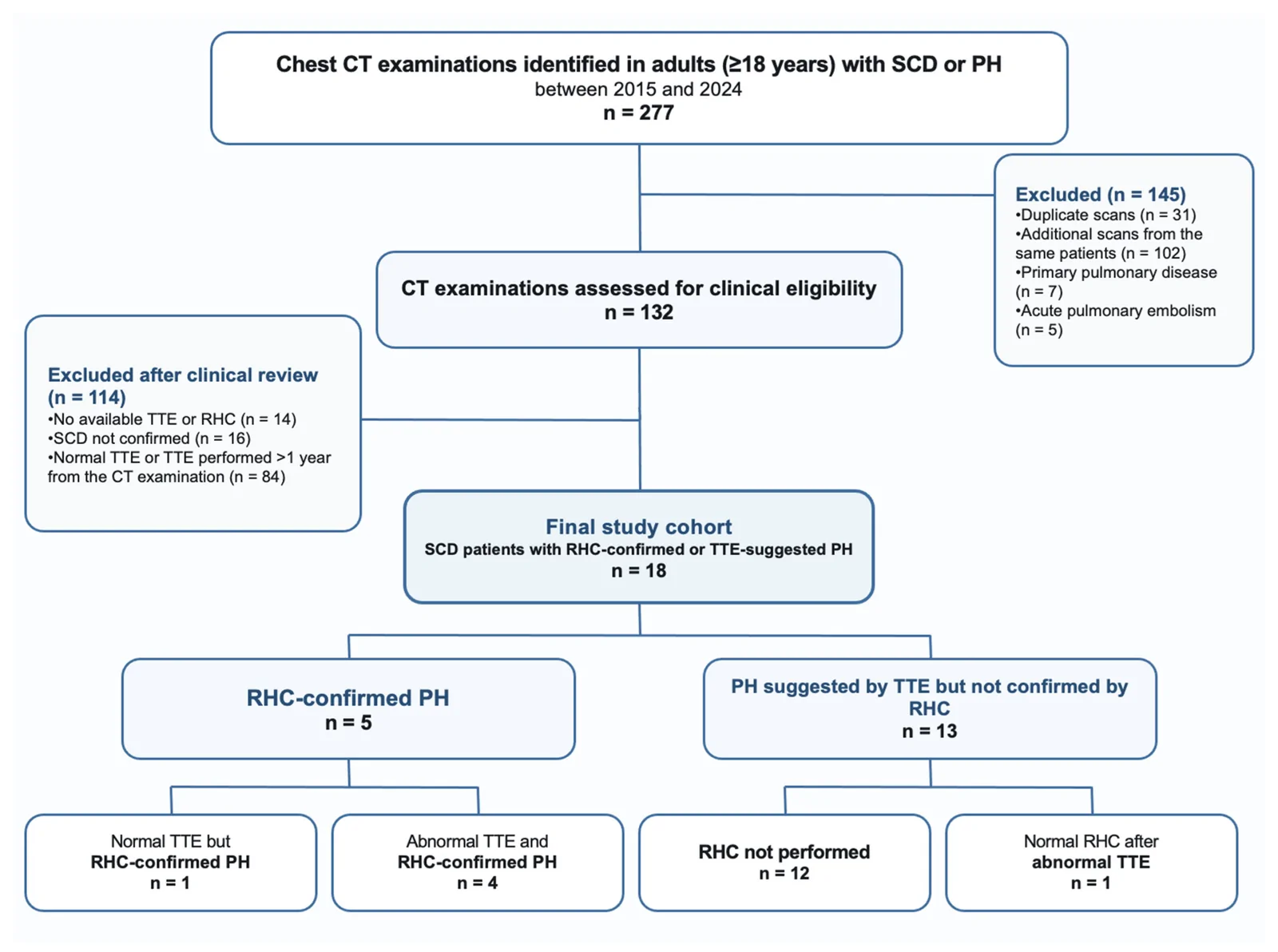
Study selection flowchart.

Eighteen patients were included in the final analysis. Five had hemodynamically confirmed PH by RHC; the remaining 13 patients were not RHC-confirmed and were analyzed as having echocardiographically suggested PH. Among the five RHC-confirmed patients, four also had abnormal TTE findings, and one had a normal TTE. Among the 13, twelve had not undergone RHC, and one had a normal RHC despite an abnormal TTE (Figure 1).

The cohort had a mean age of 35.9 ± 10.8 years at the time of CT, with ages ranging from 21 to 58 years. Gender distribution was split equally. Among the CT modalities, CTPA was the most frequently used investigation, performed in 14 patients (77.8%), while HRCT Chest and non-contrast CT Chest were utilized less often, with 3 (16.7%) and 1 (5.6%) patients, respectively. Of the 18 patients, 10 demonstrated at least one qualifying CT feature and were therefore included in the study-defined CT severity classification. Based on TTE findings, most patients had an intermediate probability of PH (11 patients, 64.7%), followed by high probability in 5 patients (29.4%), whereas only 1 patient (5.9%) had a low probability of PH (Table 1).

When severity was compared across modalities, mild PH predominated on both CT and TTE. The two modalities diverged at the severe end of the spectrum: TTE identified a greater proportion of severe cases, while CT tended to classify more patients as having mild or moderate disease (Table 2).

Main pulmonary artery enlargement (PAD ≥ 30 mm) was present in 8 of 18 patients (44.4%). The mean PAD was 2.69 ± 0.42 cm in patients evaluated by TTE alone (*n* = 13) and 3.61 ± 0.58 cm in the four patients evaluated by both TTE and RHC. Enlargement was distributed across subgroups: all four patients evaluated by both TTE and RHC met the ≥30 mm threshold, as did 4 of the 13 evaluated by TTE alone. The RHC-confirmed subgroup with a normal TTE included a single patient (*n* = 1), with a PAD of 2.25 cm; therefore, this value represents an individual measurement rather than a group estimate and should not be used for meaningful comparison with the other diagnostic subgroups. The overall mean PAD was 2.90 ± 0.60 cm (*n* = 18) (Table 3).

**Figure 2:**
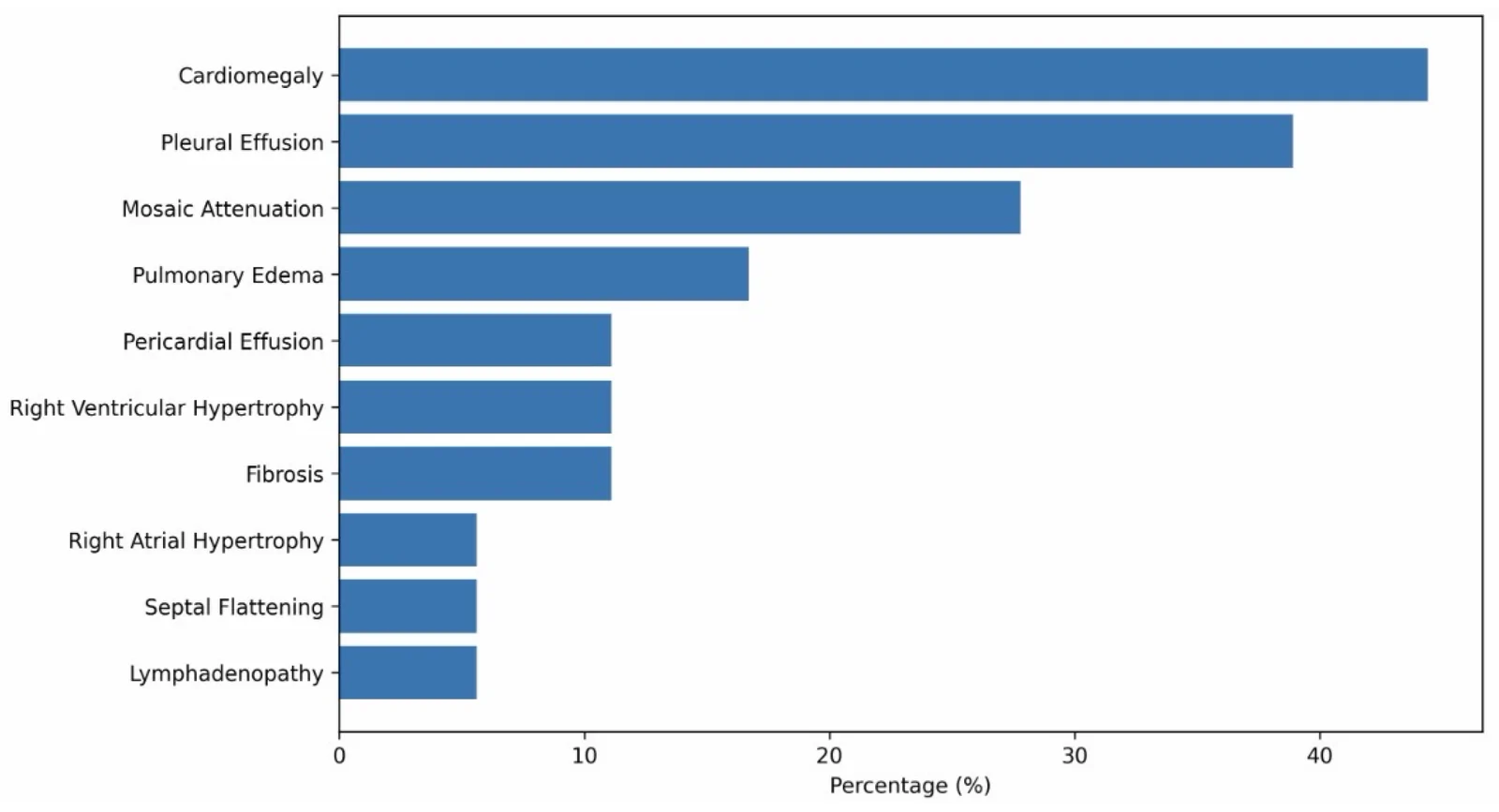
Frequency of individual CT findings in the overall study cohort (N = 18). Values are presented as *n*/N (%), with N = 18 as the denominator.

Figure 2 presents the frequency of individual CT findings in the entire study cohort (N = 18). Cardiomegaly was the most frequent finding, occurring in eight patients (8/18, 44.4%), followed by pleural effusion in seven (7/18, 38.9%) and mosaic attenuation in five (5/18, 27.8%). In contrast to the CT severity analysis, all 18 patients were included when calculating the frequencies presented in Figure 2.

Of the 18 patients included, 11 (61.1%) had mild PH, 2 (11.1%) had moderate PH, and 5 (27.8%) had severe PH. Using Fisher’s exact test with FDR correction across the eleven comparisons in Table 4 (see Section Statistical Analysis), no individual CT feature reached statistical significance, including RV hypertrophy (identified in two patients—one moderate, one severe; exact *p* = 1.00) and septal flattening (identified in one patient with severe PH; exact *p* = 0.39). The presence of any CT sign of PH increased with severity (36.4% of mild, 50.0% of moderate, and 100.0% of severe patients; exact *p* = 0.042), consistent with the Kendall’s tau-b correlation reported below, but this association was no longer statistically significant after FDR correction (q = 0.33) and should be interpreted as exploratory (Table 4).

CT signs of PH were significantly associated with PH severity using Kendall’s tau-b (τ = −0.515, *p* = 0.028; *n* = 18) (Table S1); the same association assessed by Fisher’s exact test across the three severity categories was consistent (exact *p* = 0.042, Table 4), although it did not remain significant after FDR correction (q = 0.33, Table 4).

A weak positive correlation was observed between PAD and echocardiographic-based PH probability (Kendall’s τ = 0.295). However, the association was not statistically significant (*p* = 0.151), indicating that PAD alone was not significantly associated with TTE- based PH probability in this cohort (N = 17) (Table S2).

When CT features were examined specifically as predictors of severe disease, we used dedicated 2 × 2 Fisher’s exact tests (severe vs. non-severe) with exact odds ratios and 95% confidence intervals, since the CT-based comparison involves only a single severe patient against nine non-severe patients. Neither septal flattening (exact *p* = 0.10; OR not estimable, 95% CI 0.23–∞) nor RV hypertrophy (exact *p* = 0.20; OR not estimable, 95% CI 0.10–∞) reached statistical significance on CT-based assessment once tested this way, and none of the five CT features examined had an FDR-adjusted q-value below 0.50. On TTE-based assessment (5 severe vs. 13 non-severe), mosaic attenuation was the most frequent feature among patients with severe PH (60%) and showed the strongest, though still non-significant, association (exact *p* = 0.099; OR 7.06, 95% CI 0.49–147); no CT feature reached statistical significance against TTE-defined severe disease by exact testing (Table 5). Given that they rest on a single severe case, the CT-based comparisons in particular should be read as descriptive rather than as evidence of a predictive association.

## 4. Discussion

In this retrospective cohort of adults with SCD and either echocardiographic suspicion of PH or RHC-confirmed PH, CT demonstrated pulmonary vascular, cardiac, and parenchymal abnormalities that may contribute to the structural assessment of PH in this population. Prior imaging studies have reported that enlargement of the main pulmonary artery on CT can support the presence of PH, particularly when the diameter approaches or exceeds commonly used thresholds, although this measurement should not be interpreted as a stand-alone diagnostic marker [25,26,37,38]. In this context, pulmonary artery enlargement in our cohort is consistent with previously described CT patterns of PH and may reflect pulmonary vascular remodeling among patients with SCD-associated PH.

In our cohort, cardiomegaly (44.4%), pleural effusion (38.9%), and mosaic attenuation (27.8%) were the most frequently observed CT findings. These findings are consistent with previously reported CT manifestations of PH and may reflect chronic cardiopulmonary changes associated with the disease [26,28].

Cardiomegaly and right-sided cardiac changes may reflect the effect of elevated pulmonary pressures on cardiac structure, while mosaic attenuation may represent heterogeneous pulmonary perfusion related to pulmonary vascular disease. Similar cardiopulmonary imaging abnormalities have been described in patients with SCD-associated PH, supporting the value of CT as a complementary tool for identifying structural and parenchymal changes that may not be fully characterized by echocardiography alone [22,28].

A statistically significant correlation was observed between CT signs of PH and PH severity using Kendall’s tau-b (τ = −0.515, *p* = 0.028), and the same association assessed by Fisher’s exact test across severity categories was consistent (exact *p* = 0.042; Table 4) but was no longer statistically significant after FDR correction (q = 0.33). Septal flattening and RV hypertrophy were numerically more frequent among patients with severe PH, but neither association reached statistical significance once tested with Fisher’s exact method appropriate for these small cell counts (exact *p* = 0.39 for septal flattening and *p* = 1.00 for RV hypertrophy; Table 4). In any case, septal flattening and RV hypertrophy are well-established imaging manifestations of PH regardless of the underlying etiology and are not specific to patients with SCD [25,26]. This is important when interpreting the present findings, as CT features such as septal flattening and RV hypertrophy may indicate increased right-sided pressure load, but they cannot determine the underlying cause of PH. Severe CT-based PH was identified in only one patient, and severe TTE-based PH in five; the associations involving severe PH in Table 5 should therefore be interpreted as descriptive rather than confirmatory.

Comparison between CT-based and TTE-based severity assessment demonstrated that CT tended to slightly overestimate mild and moderate PH while underestimating severe cases. This discrepancy may reflect the structural nature of CT findings compared with the hemodynamic information obtained through echocardiographic assessment. The discordance between CT and TTE in this cohort highlights an important diagnostic challenge. CT provides anatomic and parenchymal information, whereas TTE estimates the probability of PH based on functional and hemodynamic parameters. Previous studies in SCD have shown that TTE estimates may not always correspond precisely with invasive hemodynamic measurements, and that Doppler echocardiography may lead to misclassification when compared with RHC [23,24]. Therefore, within the context of imaging-based assessment, CT and TTE should be regarded as complementary rather than interchangeable modalities. Intermediate echocardiographic PH probability was the most frequent category among patients with available probability assessment in our cohort (11/17, 64.7%). This finding should be interpreted descriptively and may reflect the characteristics and clinical selection of the study cohort rather than the diagnostic performance of TTE.

The relationship between cohort-ascertainment category and echocardiography-based PH probability further supports this interpretation. Among patients with available echocardiographic probability assessment, ascertainment category was significantly associated with PH probability on TTE (*p* = 0.001). Most patients included because of echocardiographic suspicion without RHC confirmation had an intermediate probability of PH, while all patients with both abnormal TTE and RHC-confirmed PH had a high probability of PH. This pattern likely reflects clinical selection, where patients with higher echocardiographic probability or greater clinical concern were more likely to undergo catheterization. However, this finding should not be interpreted as a measure of diagnostic sensitivity or specificity because RHC was not performed in all patients. Given that RHC remains the reference standard for confirming PH and characterizing pulmonary hemodynamics, future studies with systematic RHC data are needed to better assess concordance between CT, TTE, and RHC [23,36].

Overall, CT may help identify pulmonary artery enlargement, cardiac remodeling, and parenchymal abnormalities that can support clinical suspicion and guide further evaluation of SCD-associated PH. However, our findings also demonstrate the limitations of relying on structural imaging alone, particularly for severity classification. A multimodal approach integrating CT findings with echocardiography and selective RHC remains important for a more complete evaluation of PH in patients with SCD. Although CT provides rapid, high-resolution assessment of the pulmonary vasculature, lung parenchyma, and cardiovascular structures, its potential diagnostic benefit should be balanced against exposure to ionizing radiation, particularly when repeated examinations are anticipated [26]. Therefore, CT should be used selectively as a complementary imaging modality when the expected anatomic or etiologic information is likely to contribute meaningfully to clinical evaluation.

This study has some limitations. First, its retrospective design relied on previously recorded clinical and imaging data, which limited the completeness and consistency of several variables. Second, the relatively small cohort reduced statistical power, limited the ability to detect meaningful associations, and precluded reliable multivariable regression analyses. Third, CT protocols were heterogeneous across the study period, including contrast-enhanced and non-contrast acquisitions with varying slice thickness and reconstruction parameters, which may have affected the detection and reproducibility of the vascular and parenchymal findings assessed. Furthermore, RHC data were available for only a minority of patients, and several hemodynamic parameters were missing or inconsistently documented. Because most PH cases were identified based on TTE alone, and given the limited specificity of echocardiographic estimates in patients with SCD, the cohort may have included individuals without hemodynamically confirmed PH. Consequently, direct comparison with the reference standard and calculation of diagnostic sensitivity and specificity were not feasible, and the study remains susceptible to verification bias. In addition, the intervals between CT, TTE, and RHC examinations were not consistently documented, introducing potential temporal variability, particularly given the dynamic hemodynamic status that may occur in patients with SCD.

Several SCD-related clinical and treatment variables that could influence pulmonary artery diameter and other cardiopulmonary CT findings were also not systematically available or temporally aligned with the CT examinations. These included SCD genotype, contemporaneous hemoglobin level and anemia severity, markers of hemolysis, hydroxyurea therapy, history of acute chest syndrome or thromboembolic disease, transfusion status, and relevant cardiac comorbidities. Therefore, adjustment for these potential confounders was not possible. Furthermore, the clinical indications for CT examinations were not systematically documented. Because inclusion required the availability of CT imaging together with TTE and/or RHC data, referral and selection bias cannot be excluded, as patients undergoing CT may represent a clinically selected population with greater cardiopulmonary concern, thereby limiting the generalizability of the findings to the broader SCD population. Although the inclusion criteria specified confirmation of PH by TTE and/or RHC, the retrospective nature of the study meant that uniform confirmation using both modalities could not be achieved across the cohort.

Given the small cohort and the number of features tested, associations were assessed using exact tests with FDR correction (see Section Statistical Analysis). After FDR correction, no individual CT feature retained a statistically robust association with PH severity, and the observed associations should be considered exploratory and hypothesis-generating rather than confirmatory, particularly in the context of the small sample size. Finally, the PH severity grading approach used in this study was exploratory and has not undergone external validation. It should therefore not be interpreted as a validated CT-based PH severity classification system or as equivalent to established TTE- or hemodynamic-based grading methods. Although the severity thresholds were applied uniformly, the underlying RVSP values and the original CT reports were generated by multiple echocardiographers and radiologists over the study period, representing an additional source of variability in the severity classifications. Future prospective studies with larger cohorts, standardized timing of CT, TTE, and RHC, systematic collection of SCD-related clinical variables, and broader use of hemodynamic confirmation are needed to validate these findings.

Despite these limitations, the study has important strengths. It focuses on CT imaging findings in patients with SCD-associated PH, an area with limited radiologic data. It also integrates CT findings with available echocardiographic and catheterization data, providing a practical real-world assessment of how these modalities may complement each other. In addition, the study highlights specific CT features associated with PH severity, while emphasizing that these findings are not specific to SCD and should be interpreted in the broader clinical context. Future suggestions include a similar prospective, multicenter study.

## 5. Conclusions

In conclusion, CT demonstrated several vascular, cardiac, and parenchymal abnormalities in adult patients with SCD-associated PH, including pulmonary artery enlargement, cardiomegaly, pleural effusion, and mosaic attenuation. However, these findings are not specific for PH related to SCD, and this study was restricted to patients with SCD; we make no claim regarding CT’s diagnostic performance in other forms of pulmonary hypertension, and extrapolation to other PH etiologies would require dedicated comparative studies. Associations between individual CT features and PH severity should be regarded as exploratory: none remained statistically robust after appropriate exact testing and correction for multiple comparisons (see Section 3). Overall, CT may provide useful complementary structural information in the assessment of PH in patients with SCD; however, CT should be integrated with, and not substituted for, TTE and RHC when indicated.

## Figures and Tables

**Table 1:** Clinical and demographic characteristics of patients evaluated for suspected or confirmed PH.

Variable	N (%)	
Age (years) Mean ± standard deviation (SD)	35.9 ± 10.8	
Gender		
Male	9	50
Female	9	50
Total	18	100
Body mass index (BMI)		
Underweight (<18.5 kg/m^2^)	1	5.6
Normal (18.5–24.9 kg/m^2^)	10	55.6
Overweight (25.0–29.9 kg/m^2^)	7	38.9
Total	18	100
PH ascertainment category		
TTE	13	72.2
RHC	1	5.6
TTE and RHC	4	22.2
Total	18	100
Signs of PH on CT		
Yes	10	55.6
No	8	44.4
Total	18	100
PH Probability on TTE		
Low	1	5.9
Intermediate	11	64.7
High	5	29.4
Total *	17	100

* Echocardiographic PH probability was available for 17 of 18 patients; one patient was diagnosed by RHC alone and did not have available echocardiographic probability data.

**Table 2:** Comparison of pulmonary hypertension (PH) severity distribution based on CT and TTE findings.

PH Severity	Based on CT	Based on TTE
	***n*** **(%)**	***n*** **(%)**
Mild	7 (70)	11 (61.1)
Moderate	2 (20)	2 (11.1)
Severe	1 (10)	5 (27.8)
Total	10 (100)	18 (100)

CT severity was classified based on the study-defined feature count, whereas TTE severity was classified according to RVSP thresholds (see Section 2).

**Table 3:** Pulmonary artery diameter on CT according to PH ascertainment category.

PH Ascertainment Category	*n*	PAD (cm), Mean ± SD
TTE	13	2.69 ± 0.42
RHC	1	2.25 *
TTE and RHC	4	3.61 ± 0.58
Overall cohort	18	2.90 ± 0.60

* The RHC-only subgroup included a single patient (*n* = 1); therefore, the reported PAD represents an individual measurement, and an SD could not be calculated.

**Table 4:** Association between CT imaging features and severity of PH.

CT Feature	Mild (N = 11), *n*/N (%)	Moderate (N = 2) *n*/N (%)	Severe (N = 5) *n*/N (%)	Exact *p*-Value	FDR q-Value
Pulmonary edema	3/11 (27.3%)	0/2 (0.0%)	0/5 (0.0%)	0.663	0.912
Pleural effusion	6/11 (54.5%)	1/2 (50.0%)	0/5 (0.0%)	0.119	0.326
RV hypertrophy	1/11 (9.1%)	0/2 (0.0%)	1/5 (20.0%)	1.000	1.000
RA hypertrophy	0/11 (0.0%)	0/2 (0.0%)	1/5 (20.0%)	0.389	0.611
Septal flattening	0/11 (0.0%)	0/2 (0.0%)	1/5 (20.0%)	0.389	0.611
Mosaic attenuation	1/11 (9.1%)	1/2 (50.0%)	3/5 (60.0%)	0.067	0.326
Cardiomegaly	5/11 (45.5%)	1/2 (50.0%)	2/5 (40.0%)	1.000	1.000
Pericardial effusion	1/11 (9.1%)	0/2 (0.0%)	1/5 (20.0%)	1.000	1.000
Lymphadenopathy	0/11 (0.0%)	1/2 (50.0%)	0/5 (0.0%)	0.111	0.326
Fibrosis	1/11 (9.1%)	1/2 (50.0%)	0/5 (0.0%)	0.281	0.611
Signs of PH on CT	4/11 (36.4%)	1/2 (50.0%)	5/5 (100.0%)	0.042	0.326

Values are presented as *n*/N (%), where *n* represents the number of patients with the specified CT finding, and N represents the total number of patients within each PH severity category, based on TTE/RHC-defined severity (mild, N = 11; moderate, N = 2; severe, N = 5). Percentages were calculated within each PH severity group. *p*-values are from Fisher’s exact test (two-sided and unadjusted; see Section Statistical Analysis). The q-value is the FDR-adjusted *p*-value across all eleven comparisons in this table. No comparison reached significance (q < 0.05).

**Table 5:** Association of CT imaging features with severe PH based on CT and TTE assessment.

CT Feature	Based on CT, *n* (%)—1 Severe/9 Non-Severe	Exact *p* (CT)	OR (95% CI), CT	Based on TTE, *n* (%)—5 Severe/13 Non-Severe	Exact *p* (TTE)	OR (95% CI), TTE
Mosaic Attenuation	1 (100.0%)	1.000	not estimable (0.03–∞)	3 (60.0%)	0.099	7.06 (0.49–147)
Septal Flattening	1 (100.0%)	0.100	not estimable (0.23–∞)	1 (20.0%)	0.278	not estimable (0.07–∞)
Cardiomegaly	0 (0.0%)	1.000	0 (0–39)	2 (40.0%)	1.000	0.79 (0.05–9.6)
RV Hypertrophy	1 (100.0%)	0.200	not estimable (0.10–∞)	1 (20.0%)	0.490	2.79 (0.03–255)
RA Hypertrophy	0 (0.0%)	1.000	0 (0–351)	1 (20.0%)	0.278	not estimable (0.07–∞)

Because the CT-based severe group contains a single patient and the TTE-based severe group contains five, each row above is a dedicated 2 × 2 Fisher’s exact test (severe vs. non-severe) with exact odds ratios and 95% confidence intervals; “not estimable” indicates a zero cell that leaves the odds ratio mathematically undefined. Across all ten comparisons in this table, FDR-adjusted q-values were ≥0.50; these findings should be read as descriptive rather than as evidence of a predictive association. The original table (based on chi-square-derived *p*-values, without odds ratios) is reproduced for comparison in the accompanying Response to Reviewers letter.

## Data Availability

The datasets generated and/or analyzed during the current study are not publicly available due to patient privacy; however, they are available from the corresponding author upon reasonable request.

## Supplementary Materials

The following supporting information can be downloaded at: https://www.mdpi.com/article/10.3390/jcm15197611/s1, Table S1: Correlation Between CT Signs of Pulmonary Hypertension and PH Severity, Table S2: Correlation Between PAD and TTE-Based Probability of PH, Table S3: Association Between Method of Diagnosis and TTE-Based Probability of PH.

## Abbreviations

The following abbreviations are used in this manuscript:

CT	Computed Tomography
CTPA	Computed Tomography Pulmonary Angiography
CXR	Chest X-Ray
DECT	Dual-Energy Computed Tomography
ESC	European Society of Cardiology
FDR	False Discovery Rate
HbS	Hemoglobin S
HRCT	High-Resolution Computed Tomography
mPAP	Mean Pulmonary Artery Pressure
NO	Nitric Oxide
PA	Pulmonary Artery
PAD	Pulmonary Artery Diameter
PAH	Pulmonary Arterial Hypertension
PH	Pulmonary Hypertension
RA	Right Atrium
RHC	Right Heart Catheterization
RV	Right Ventricle
RVSP	Right Ventricular Systolic Pressure
SCD	Sickle Cell Disease
SCT	Sickle Cell Trait
SD	Standard Deviation
SPECT	Single-Photon Emission Computed Tomography
TR	Tricuspid Regurgitation
TRV	Tricuspid Regurgitation Jet Velocity
TTE	Transthoracic Echocardiography
