# Supplementary material for: CT Features of Sickle Cell Disease-Associated Pulmonary Hypertension
Source: J Clin Med. 2026 Oct 1;15(19):7611. doi: 10.3390/jcm15197611 (PMC13666314; doi:10.3390/jcm15197611)
Supplement: Supplementary file 1 [file jcm-15-07611-s001.zip › jcm-4486585-supplementary.pdf]

Table S1. Correlation Between CT Signs of Pulmonary Hypertension and PH Severity.

		PH Severity
Signs of PH on CT	Kendall tau b Correlation	-0.515*
	Sig. (2-tailed)	0.028
	N	18

Table S2. Correlation Between Pulmonary Artery Diameter and TTE-Based Probability of Pulmonary Hypertension.

		PH Probability on TTE (based on the TR Jet)
Pulmonary artery diameter (cm)	Kendall tau b Correlation	0.295
	Sig. (2-tailed)	0.151
	N	17

Table S3. Association Between Method of Diagnosis and TTE-Based Probability of Pulmonary Hypertension (PH)

Variables			PH Probability on TTE (based on the TR Jet)			Total	p value
			Low	Intermediate	High		
Method of diagnosing PH	TTE	Count	1	11	1	13	0.001*
		percentage	7.7%	84.6%	7.7%	100.0%	
	TTE + RHC	Count	0	0	4	4	
		percentage	0.0%	0.0%	100.0%	100.0%	
Total		Count	1	11	5	17	
		percentage	5.9%	64.7%	29.4%	100.0%	

* p value < 0.05 indicates significance obtained at 5% level (using Chi-square test of association).
